# NeuroART: Real-Time Analysis and Targeting of Neuronal Population Activity during Calcium Imaging for Informed Closed-Loop Experiments

**DOI:** 10.1523/ENEURO.0079-24.2024

**Published:** 2024-10-10

**Authors:** Zac Bowen, Dulara De Zoysa, Kelson Shilling-Scrivo, Samira Aghayee, Giorgio Di Salvo, Aleksandr Smirnov, Patrick O. Kanold, Wolfgang Losert

**Affiliations:** ^1^Institute for Physical Science and Technology, University of Maryland, College Park, Maryland 20742; ^2^Fraunhofer USA Center Mid-Atlantic, Riverdale, Maryland 20737; ^3^Fischell Department of Bioengineering, University of Maryland, College Park, Maryland 20742; ^4^Department of Anatomy and Neurobiology, University of Maryland School of Medicine, Baltimore, Maryland 21230; ^5^Department of Biology, University of Maryland, College Park, Maryland 20742; ^6^Department of Biomedical Engineering, Johns Hopkins University, Baltimore, Maryland 20215; ^7^Kavli NDI, Johns Hopkins University, Baltimore, Maryland 20215

**Keywords:** auditory cortex tuning, holographic optogenetics, neuronal population activity, neuronal receptive fields, real-time analysis, two-photon calcium imaging

## Abstract

Two-photon calcium imaging allows for the activity readout of large populations of neurons at single cell resolution in living organisms, yielding new insights into how the brain processes information. Holographic optogenetics allows us to trigger activity of this population directly, raising the possibility of injecting information into a living brain. Optogenetic triggering of activity that mimics “natural” information, however, requires identification of stimulation targets based on real-time analysis of the functional network. We have developed NeuroART (Neuronal Analysis in Real Time), software that provides real-time readout of neuronal activity integrated with downstream analysis of correlations and synchrony and of sensory metadata. On the example of auditory stimuli, we demonstrate real-time inference of the contribution of each neuron in the field of view to sensory information processing. To avoid the limitations of microscope hardware and enable collaboration of multiple research groups, NeuroART taps into microscope data streams without the need for modification of microscope control software and is compatible with a wide range of microscope platforms. NeuroART also integrates the capability to drive a spatial light modulator (SLM) for holographic photostimulation of optimal stimulation targets, enabling real-time modification of functional networks. Neurons used for photostimulation experiments were extracted from Sprague Dawley rat embryos of both sexes.

## Significance Statement

We have developed a software platform, Neuronal Analysis in Real Time (NeuroART), which addresses the growing need in neuronal imaging studies for real-time analysis capabilities and has unique capabilities when compared with other recently developed software (Giovannucci et al., 2017; Mitani and Komiyama, 2018; Zhang et al., 2018; Giovannucci et al., 2019; Sheng et al., 2022). NeuroART stands out in its real-time inclusion of functional network analysis, correlation analysis, synchrony analysis, holographic optogenetic photostimulation, and integration of sensory information metadata. Furthermore, this tool enables experimenters to assess data quality in real time. With these unique features and its demonstrated ability to work with several widely used microscope platforms, NeuroART is poised to enable novel closed-loop model-guided experiments.

## Introduction

Multiphoton laser scanning microscopy of cytoplasmic calcium concentrations is a powerful tool in modern neuroscience, enabling researchers to observe the temporal dynamics of large populations of neurons with single-cell resolution. Neurons can be labeled with exogenous and genetically encoded calcium indicators that produce time series of neuronal activity for each neuron in an imaging field of view (FOV; Greenberg et al., 2008; Kerr and Denk, 2008; Dombeck and Tank, 2014). Countless studies have used two-photon (2P) excitation fluorescence imaging to great effect providing groundbreaking insights into the functionality of the brain (Svoboda et al., 1997; Ohki et al., 2005; Wang et al., 2006; Maeda et al., 2014).

Recent advances in holographic optogenetics make it possible to target groups of cells for stimulation, e.g., to mimic the neuronal activity seen during sensory perception (Gill et al., 2020; Paul et al., 2023; Kang et al., 2024). These studies showed that injecting information into neuronal networks requires coordinated input into groups of functionally linked neurons (Gill et al., 2020). However, identification of functional groups of neurons is usually done after the end of an experiment, since 2P imaging data is inherently noisy and requires significant preprocessing and analysis to produce interpretable signals (Oheim et al., 2001; Mitani and Komiyama, 2018; Griffiths et al., 2020). Thus, the typical cadence within a neuroimaging study is to conduct 2P imaging and then analyze the data after the experiment has concluded.

Utilizing the neuronal population readout from these 2P image analysis pipelines and performing subsequent downstream analysis, one can begin to explore questions about the function of the brain such as (1) which neurons are most active, (2) which neurons are most correlated, (3) what the sensory receptive field of each neuron is, and (4) which neurons are encoding behavioral choice. Having an answer to these four questions in real time would facilitate rapid insight into the responsiveness of a neuronal population to stimuli, on-the-fly measurement of the functional relationships and tuning properties of a neuronal population, and closed-loop experiments with informed optogenetic stimulation including new model-based experimental paradigms. However, most of the downstream analysis of neuroimaging data is done offline in existing software packages without real-time capabilities. There exists a growing need for user-friendly software that can go beyond preprocessing and perform downstream analysis and photostimulation in real time during a 2P imaging experiment.

**Figure 1.**
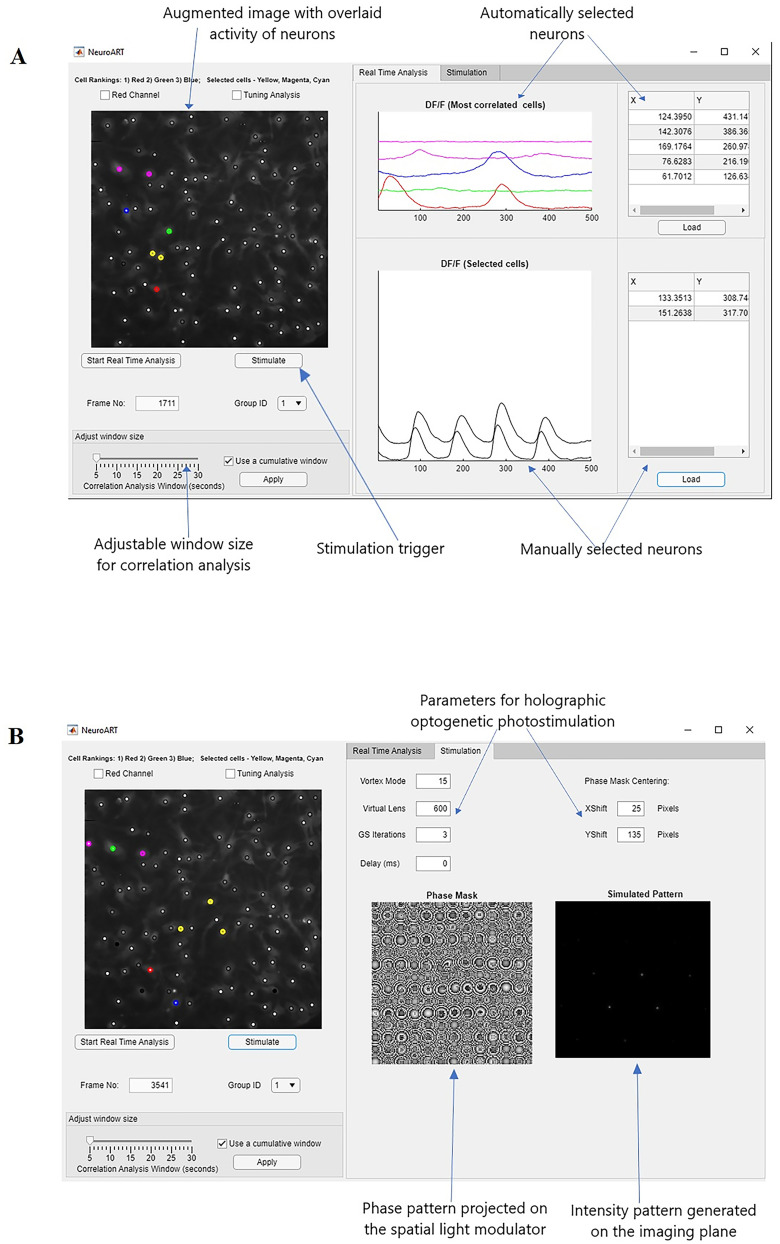
NeuroART GUI for analysis and stimulation. ***A***, GUI for real-time analysis. Key features highlighted include the augmented image with overlaid neuronal activity, automatically identified and manually selected neurons to closely monitor *ΔF*/*F*_0_ traces, and the photostimulation trigger for optogenetic stimulation. ***B***, Separate tab for holographic stimulation. Both the phase mask (phase pattern projected on the SLM) and the simulated intensity patterns are displayed for the convenience of the user.

We have developed software called NeuroART (Neuronal Analysis in Real Time) that processes and analyzes 2P neuroimaging data in real time, enabling observation and quantification of neuronal population dynamics during an in vitro, acute slice, or live-animal experiment. Our real-time analysis application runs via an easy-to-use graphical user interface (GUI) in MATLAB (Fig. 1). NeuroART taps into existing data streams of microscopes and allows us to rapidly analyze every image as it is acquired, integrate other experimental information (e.g., stimulus parameters), and thus quantify the functional properties of all imaged neurons while the experiment is in progress. Furthermore, the photostimulation module of NeuroART enables holographic optogenetic stimulation of neurons that are identified as potential stimulation targets based on real-time analysis.

**Figure 2.**
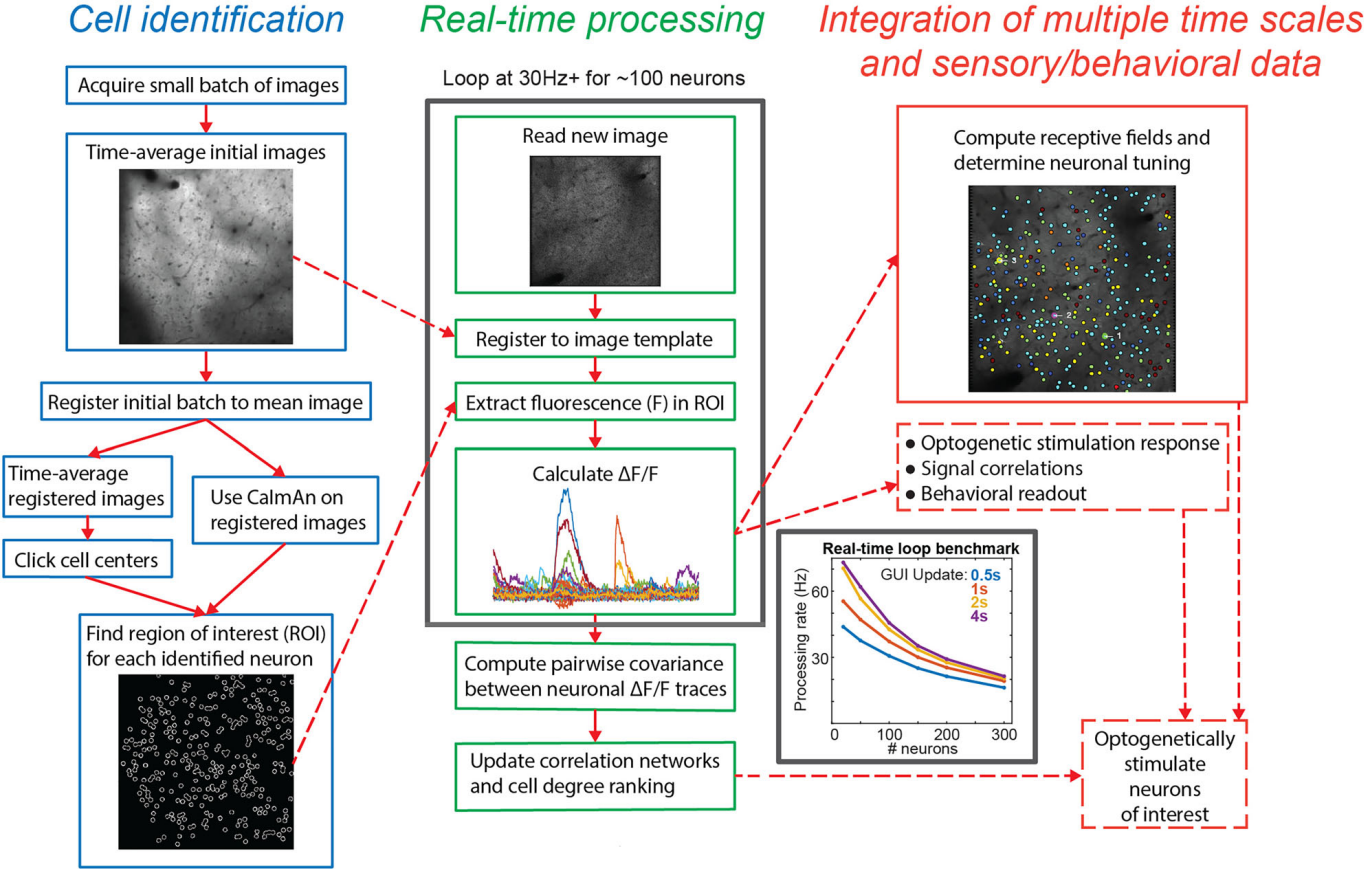
Analysis workflow. Left, Identifying cell ROIs by manual clicking, automatic detection via CaImAn, or loading previously identified cell coordinates. Middle, Real-time processing on each acquired image (30 frames per second for ∼150 neurons, higher rates for fewer neurons, rate dependence shown on inset plot). Right, Analysis of signal correlations, synchrony, and behavioral readout on multiple timescales spanning from updating at the image acquisition rate to minutes.

NeuroART is focused on downstream analysis and can work on a separate computer with the most popular microscope platforms, making it well suited for team collaborations among multiple labs and collaborations between experimental and theory labs. This focus distinguishes it from the rapidly growing group of 2P calcium image analysis pipelines (Pachitariu et al., 2017; Zhou et al., 2018; Giovannucci et al., 2019; Cantu et al., 2020; Lee et al., 2020; Guarnieri et al., 2021; Sheng et al., 2022). NeuroART stands out in its real-time inclusion of advanced functional network analysis, correlation analysis, and informed real-time holographic optogenetic stimulation capabilities which are not available in any of the recently developed software tools for real-time analysis (Giovannucci et al., 2017; Prsa et al., 2017; Mitani and Komiyama, 2018; Zhang et al., 2018; Giovannucci et al., 2019; Griffiths et al., 2020; Sheng et al., 2022). The overall workflow of NeuroART is illustrated in Fig. 2, which will be discussed in detail in the results section.

## Materials and Methods

### Experimental procedures for example datasets

All procedures were approved by the University of Maryland Institutional Animal Care and Use Committee (IACUC). Figures for in vivo experiments are based on two-photon imaging performed on one adult mouse (postnatal day 185) expressing genetically encoded calcium indicator GCaMP6s. Mouse was F1 offspring of Thy1-GCaMP6s (JAX: 024275) mice crossed with CBA/CaJ mice (JAX: 000654) and was implanted with a cranial window centered above the auditory cortex. Imaging was performed as described previously (Francis et al., 2018; Bowen et al., 2019; Liu et al., 2019) on a rotatable microscope (Bergamo II series, B248, Thorlabs) using a pulsed femtosecond Ti:Sapphire 2P laser (Vision-S, Coherent) using ThorImage LS and ThorSync software (version 3.1). Imaging was performed at 940 nm excitation wavelength with a ∼370 µm × 370 µm FOV at a frame rate of 30 Hz. Sound stimuli consisted of sinusoidally amplitude-modulated tones played at a range of frequencies (3−45.3 kHz at half-octave spacing) and sound pressure levels (50, 60, 70 dB) with 10 trial repeats for each unique stimulus. Each tone presentation had 1 s duration with a 3 s intertrial interval.

Primary rat embryonic cortical neurons from Sprague Dawley rats were used for the in vitro experiments to demonstrate holographic optogenetic stimulation capabilities of NeuroART. Embryos of both sexes were obtained from killed pregnant rats at embryonic day of gestation 18 (E18) according to and with the approval of the University of Maryland IACUC protocol (R-JAN-18-05, R-FEB-21-04, R-JAN-24-01). Following the dissection of hippocampi and cortices, the cortices were gently triturated using a fire-polished pipette. These cells were then plated onto culture dishes that had been precoated with poly-D-lysine, which promotes neuronal cell adhesion and growth. Subsequently, the cultured cells were maintained in neurobasal media and incubated at 37°C temperature and 5% carbon dioxide. After incubation for 3 d, neuronal cells were transduced using the bicistronic lentiviral vector, pLV[Exp]-Bsd-SYN1-jGCaMP8s-P2A-ChrimsonR-ST, which provides robust coexpression of the calcium indicator (jGCaMP8s) and the opsin (stChrimsonR) used for holographic optogenetic stimulation (LaFosse et al., 2023). Neurons were incubated at 37°C temperature and 5% carbon dioxide, while doing full media swaps every 3 d. In vitro calcium imaging and holographic optogenetic photostimulation were performed 1 week after transduction.

### 2P photostimulation and imaging laser setup for in vitro experiments

**Figure 3.**
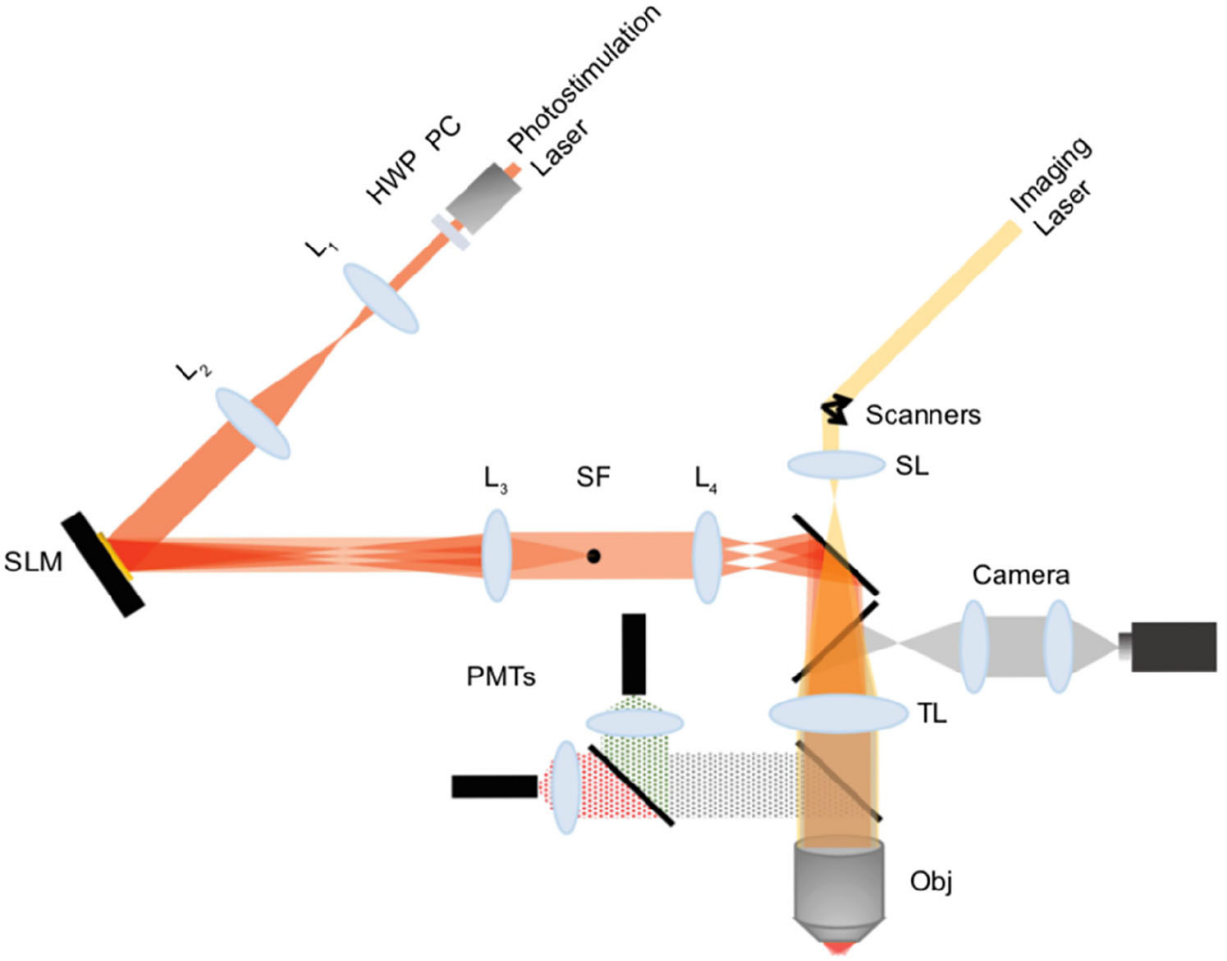
Custom-built dual beam path microscope for imaging and holographic optogenetic stimulation. A fixed laser beam of 1,064 nm is used for photostimulation, and the imaging laser is tunable within the range, 680–1,300 nm. 2P images are captured and amplified using photomultiplier tubes in parallel to simultaneous wide-field imaging through Hamamatsu ImagEM X2 EMCCD camera. (L1, L2, L3, L4, lenses; PC, Pockels cell; HWP, half wave plate; SL, scan lens; TL, tube lens; PMTs, photomultiplier tubes; Obj, objective; SLM, spatial light modulator). A 25×/1.05 NA water immersion objective and Boulder Nonlinear Systems (1,536 × 1,536) SLM with voltage overdrive and thermoelectric heating are used in our photostimulation setup.

Simultaneous imaging and photostimulation were performed using a custom-built dual beam path microscope (Fig. 3). 2P calcium images (512 × 512 pixels per frame, 30 frames per second) of in vitro neuronal cells were acquired using resonant-galvanometer raster scanning of a femtosecond-pulsed laser beam (Chameleon Discovery, Coherent, tunable range 680–1,300 nm), where the 2P fluorescence was captured using photomultiplier tubes (PMTs) and femto signal preamplifiers. A 25×/1.05 NA water immersion objective (Olympus) was used for 2P imaging and photostimulation. The genetically encoded calcium activity indicator, jGCaMP8s, was imaged at 920 nm with an FOV of 90 × 90 µm and adjustable laser power on sample, up to 40 mW. 2P calcium imaging is controlled by Vidrio ScanImage 2021, while image frames are read and analyzed by NeuroART simultaneously.

Our imaging system is also equipped with simultaneous epifluorescence imaging with a Hamamatsu cooled ImagEM X2 EMCCD camera, using a 20×/1.05 NA air objective. This wide-field imaging component was utilized to observe a larger FOV of the sample prior to 2P imaging. The excitation source for 2P photostimulation is a laser fixed at 1,064 nm (Fianium amplified fiber laser, adjustable average output, up to 5.25 W; pulse width, 160 fs; 80 MHz repetition rate). A reflective spatial light modulator (SLM; Boulder Nonlinear Systems; 15.36 × 15.36 mm active area, 1,536 × 1,536 pixels, calibrated for 1,064 nm) was used to generate beamlets for photostimulation of the neuronal cells that coexpress the stChrimsonR opsin along with the calcium indicator, jGCaMP8s. To stimulate neuronal cells transduced with stChrimsonR, 6.5 mW per cell is required for photostimulation (if a laser with low repetition rate in the range of 500 kHz is used). However, since our laser is 80 MHz (160 fs pulse width), a laser power in the range of 370 mW per cell was required. The active area of the SLM (Shane et al., 2019) was rescaled and mapped to the back aperture of the 2P microscope objective.

### Hologram computation

The Gerchberg–Saxton (GS) algorithm (Gerchberg and Saxton, 1972) was utilized to calculate phase masks (phase patterns) which are required to be projected on the SLM to generate holographic intensity patterns (Curtis et al., 2002). These computer-generated holograms modulate the phase front of the reflected laser beam to generate the desired intensity patterns for selective photostimulation of neuronal cells at objective focus. The NeuroART software generates phase masks required for photostimulation of the manually selected or automatically identified neurons and subsequently projects the phase masks on the SLM to generate holographic intensity patterns on the imaging FOV. It typically takes ∼200 ms to generate each phase mask. Once the photostimulation beam path is aligned properly, a separate phase mask optimization step is not required. NeuroART also provides an option for sequential stimulation, where the phase masks corresponding to each targeted neuron are predownloaded to the SLM driver. Once all the required phase masks are available, the SLM driver iterates through the phase masks sequentially, at the rate specified by the user, enabling photostimulation of neurons in a sequence.

### NeuroART system tests

NeuroART has been tested on a range of different imaging systems with different imaging software and image formats. NeuroART does not require changes to these imaging software packages. Instead, NeuroART is designed to read the output from these imaging systems, including imaging specifications or metadata (such as XML files), in real time. NeuroART can thus be deployed either on the same computer or a different computer than the imaging software if the files and hardware where images are stored are read-accessible to NeuroART.

Furthermore, NeuroART has an offline mode that can be used for troubleshooting, as well as for processing and analysis of previously acquired datasets. Here we list the system setups that have been tested at the time of submission, though the list will continue to grow. Specific system configurations of tests are shown in Table 1.

1. Imaging systems: Thorlabs B-Scope (rotatable Bergamo II series, B248), Bruker Ultima 2P and Ultima 2P Plus/Prairie View (there is an initial delay in processing due to Bruker software placing a lock on the current raw image file while it is being written to storage), Sutter MOM-2P with Resonant scanner/ScanImage, and Custom Sutter MOM with Thorlabs Multiphoton Resonant scanning kit/ThorImage LS.
2. Imaging software: Thorlabs ThorImage LS (v3.0, v3.1, v4.0), Bruker Prairie View (v.5.5U2, v.5.5U3), and Vidrio ScanImage 2021.
3. Readable image formats: u-int16 (ThorImage LS) and s-int16 (Prairie View) raw binary, u-int16 TIF, and u-int8 TIF.
4. Operating systems: Windows 7 Pro, Windows 10, Mac OSX (offline mode).

**Table 1.** Specifications of imaging systems where NeuroART has already been deployed

Imaging system	Imaging software	Readable image formats	Operating system, storage, data link
Thorlabs B-Scope (Bergamo II series, B248)	ThorImage LS v. 3.1	u-int16 raw binary	Windows 7 Pro, RAID6 NAS, 10Gb line
Bruker Ultima 2P	PrairieView v. 5.5U3	Series of s-int16 raw binary files converted to u-int16	Windows 7 Pro, RAID0, local TCP socket interface
Bruker Ultima 2P+	PrairieView v. 5.5U2	Series of s-int16 raw binary files converted to u-int16	Windows10, SSD, local TCP socket interface
Sutter MOM with resonant scanner	ScanImage 2021 (Vidrio Technologies)	u-int16 TIF stack converted to u-int16 raw binary	Windows10, SSD, USB3
Custom Sutter MOM with Thorlabs Multiphoton Resonant scanning kit	ThorImage LS v. 4.0	u-int16 raw binary	Windows7, RAID0, USB3
Offline Mode	N/A	u-int16 raw binary / u-int16 TIF	Windows10/ Mac OS, SSD

### 2P image analysis

2P image sequences were processed using methods previously described (Bowen et al., 2019; Bowen et al., 2020) adapted for real-time processing. Image sequences were corrected for *x*–*y* drifts and movement artifacts using discrete Fourier transform registration (Guizar-Sicairos et al., 2008) implemented in MATLAB (MathWorks). Neurons were identified manually from the average image of the motion-corrected initial sequence of the user-specified number of frames. Ring-like region of interest (ROI) boundaries were programmatically drawn based on the method described in Chen et al. (2013). Overlapping ROI pixels (due to closely juxtaposed neurons) were excluded from analysis. For each selected neuron, a raw fluorescence signal over time (*F*_soma_) was extracted by averaging across pixels from the ROI overlying the soma. For the in vivo experiments, neuropil (NP) correction was performed on the raw fluorescence of all soma ROIs (*F*_soma_; Peron et al., 2015). In short, the neuropil ROI was drawn based on the outer boundary of the soma ROI and extended from 1 pixel beyond the soma ROI outer boundary to 15 µm excluding any pixels assigned to neighboring somata. The resulting fluorescence intensity (*F*) used for analysis was calculated as *F* = *F*_soma_ − (*α* × *F*_NP_), where we use a default value of *α* = 0.7 (adjustable) to reduce fluorescence contamination from the neuropil (Peron et al., 2015). The neuropil-corrected fluorescence for each neuron was then converted to a relative fluorescence amplitude (*ΔF*/*F*_0_), where *ΔF* = (*F* − *F*_0_). *F*_0_ was estimated by using a sliding window that calculated the average fluorescence of points less than the 50th percentile during the previous 10 s window.

### Neuronal receptive fields and tuning

**Figure 4.**
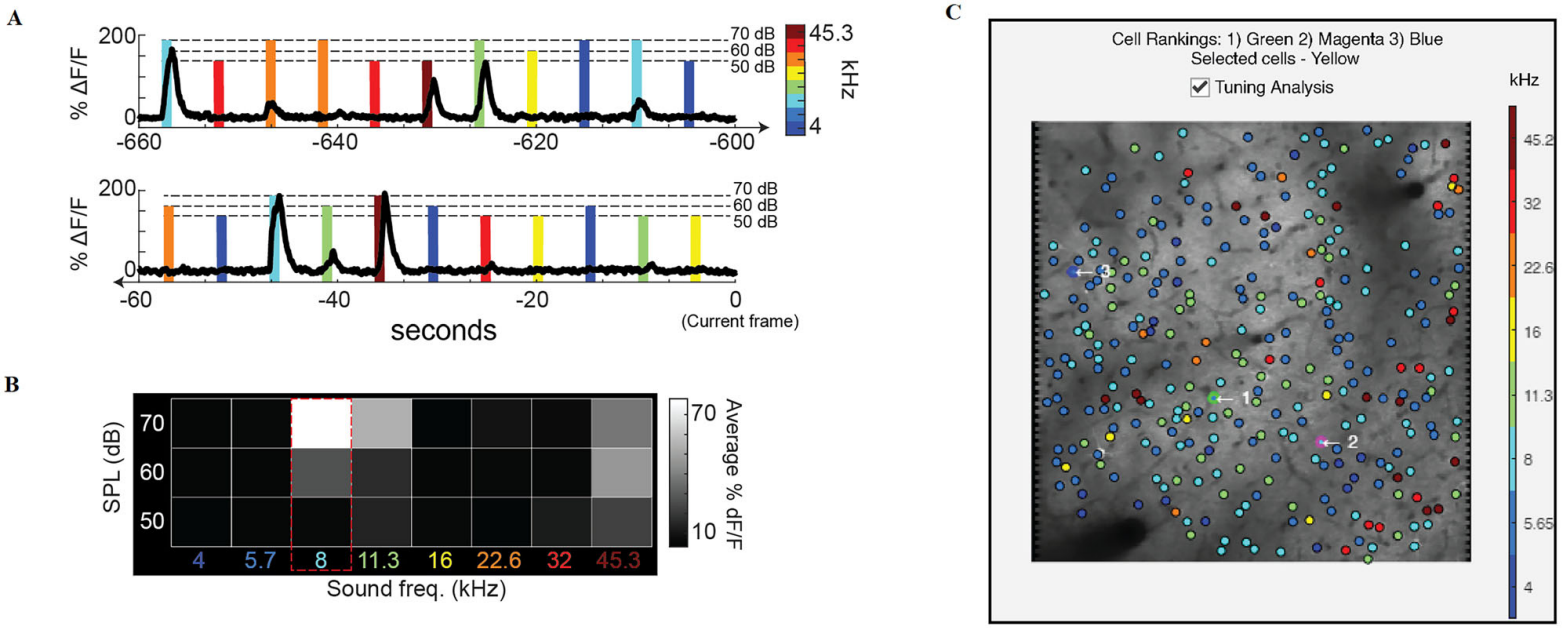
Integration of real-time stimulus information for analysis of neuronal tuning during an ongoing experiment. ***A***, Example calcium responses from a single neuron during tone presentations. Color represents the sound frequency of the stimulus and the height of the marker represents sound pressure level. ***B***, Corresponding receptive field (or frequency response area) from the example neuron where response amplitude during tone presentation is averaged over time and then organized according to the frequency and sound pressure level of the stimulus. The maximum response indicates the frequency tuning of the neuron (BF = 8 kHz in this example). ***C***, Example GUI readout during tone presentation experiment. Each neuron is colored according to its best frequency. Tuning analysis, along with this visualization, can be turned on and off during the experiment with the checkbox.

Neuron receptive fields (RFs) were determined as the average *ΔF*/*F*_0_ response to each frequency–intensity combination across all stimulus repetitions during the stimulus presentation. Best frequency (BF) for each neuron was determined as the stimulus frequency that elicited the highest average *ΔF*/*F*_0_ response at any sound level in the receptive field. Eight distinct stimulus frequencies (4, 5.7, 8, 11.3, 16, 22.6, 32, and 45.3 kHz) and three sound pressure levels (50, 60, and 70 dB) were used in the example shown in Fig. 4.

### Pairwise correlations

To assess the functional similarity of neurons, we computed correlations based on the covariance of their activity over time. Pairwise correlations were obtained for each neuronal pair by calculating the correlation coefficient of their *ΔF*/*F*_0_ utilizing MATLAB's “corrcoef” function, which performs the following:

$$\rho (A,B)=\;\frac{1}{N-1}\overset{N}{\underset{i=1}{\sum }}\;\left(\frac{A_{i}-\mu_{A}}{\sigma_{A}}\right)\left(\frac{B_{i}-\mu_{B}}{\sigma_{B}}\right),$$

where *A* and *B* represent the *ΔF*/*F*_0_ of each neuron at frame *i* out of *N* acquired imaging frames and where *µ* and *σ* represent the mean and standard deviation of *ΔF*/*F*_0_ over all frames.

### Functional networks and neuronal ranking

Functional networks are constructed by considering all pairwise correlations between neurons and then pruning them in two separate applications via (1) thresholding or (2) a topological constraint (minimum spanning tree, MST). The method employed is chosen by the user prior to entering the real-time analysis loop. The thresholding method prunes all connections below the chosen threshold amount. For the MST method, the network is pruned such that the overall weight of the graph is minimized (Bowen et al., 2024). In a response pattern of *N* nodes, the minimum spanning tree picks *N*−1 edges such that every node within the graph is connected while minimizing the total edge weight of the tree and without creating any loops within the graph. The edge weight is defined as one minus the absolute value of the pairwise correlation. This allows the weight-minimizing algorithm to pick out the most interconnected neurons. In both methods, the degree of each neuron is the number of connections to it in the functional network. The ranks of neurons are determined according to their degree within the functional network, where a higher degree corresponds to a higher rank.

### Software accessibility

The NeuroART software described in the paper and instructions to run the software are freely available online at https://github.com/losertlab/NeuroART.

## Results

### Real-time analysis software (NeuroART)

Our real-time analysis software provides an easy-to-use graphical interface and data processing pipeline integration that can be run alongside image acquisition. NeuroART provides the user with an augmented version of their imaging FOV (Fig. 1*A*, left) which is easier to visually parse than a typical live acquisition preview. The augmented FOV displays a time-averaged image of the FOV with a circle centered on each identified neuron. The center of each circle is animated with the current activity of the neuron where a brighter circle indicates a higher magnitude of activity, as measured by baseline-corrected fluorescence (*ΔF*/*F*_0_).

NeuroART allows for tracking the activity of neurons of interest in real time. During imaging, five neurons are selected automatically, with *ΔF*/*F*_0_ traces displayed (Fig. 1*A*, middle top). These are the neurons with the highest overall correlation to the rest of the population, as measured by degree in the functional correlation network (see Materials and Methods for details). At any given time, the user can manually pick neurons from these correlated neurons or other identified neurons in the FOV to be displayed in the manual section (Fig. 1*A*, middle bottom). The *ΔF*/*F*_0_ traces of manually selected neurons will remain displayed until the user decides to hide or replace them, though the app will keep track of every manually selected neuron timestamped throughout the experiment. In the augmented FOV, automatically selected cells are highlighted in different colors based on their ranking (1, red; 2, green; 3, blue; 4 and 5, magenta), and the manually selected neurons are marked with a highlighted yellow circle.

Lastly, the “Stimulation” tab (Fig. 1*B*) includes parameters for holographic optogenetic stimulation using an SLM. Once the stimulation button is pressed, the coordinates of the identified neurons for stimulation are used to generate phase masks (phase patterns to be projected on the SLM) required for holographic optogenetic stimulation. These phase masks are sent to the SLM driver in real time through the PCIe communication interface between NeuroART and the SLM. The resulting network dynamics of this perturbation can then be observed in real time by NeuroART, enabling experimenters to perform closed-loop stimulation experiments.

### Cell identification

Cell identification is an essential first step in the workflow (Fig. 2, left), so the user is given the option whether they want to manually select cells or use automatic detection algorithms provided with CaImAn (Giovannucci et al., 2019), the most widely used AI-based algorithm for Calcium image segmentation, or CITE-On (Sità et al., 2022), a recently developed fast alternative to CaImAn. Manual cell selection is performed by the experimenter by manually clicking the center of each neuron using a time-averaged image from a short initial batch of images. The automatic cell detection via CaImAn involves running the CaImAn workflow on an initial batch of images which will identify regions of interest based on activity from these initial images (implementation of CaImAn on-line version of pipeline is planned for future releases). CITE-On is a convolutional neural network-based algorithm for fast automatic cell identification in two-photon calcium imaging data.

The coordinates of neurons, identified either manually or automatically, are then passed forward to the real-time analysis loop. Manual selection requires fewer initial images than automatic selection and allows for concentration on only a subset of neurons based on experimenter's preferences. The automatic cell selection via CaImAn is expected to be significantly less biased, yet it takes more computational resources and might miss cells or have false positives. CITE-On generalizes across calcium indicators, brain regions, and acquisition parameters, while providing faster detection of cells and similar performance compared with state-of-the-art methods used for cell identification (Sità et al., 2022).

### Real-time analysis workflow

Now we introduce the workflow implemented in our current version of NeuroART. If the experimenter is employing a workflow that differs in some elements, the open-source MATLAB source code can be adjusted to the experimenter's preferred analysis approach. We chose MATLAB as a platform because it is widely used among the neuroscience research community. Furthermore, it allows users to leverage existing code and libraries written in different languages such as C/C++, Java, and Python.

Following cell identification, NeuroART enters the real-time analysis loop (Fig. 2, middle inset). The real-time loop reads each new image as it is written and leverages the average image from the cell identification step as a template to motion correct each image. Subsequently, the ROI mask is used to extract fluorescence of the relevant pixels from the image for each neuron. The values from pixels corresponding to each neuron are averaged resulting in the raw fluorescence for that time point. The raw fluorescence is then corrected for neuropil signal contamination as described in the Materials and Methods, where the fraction of neuropil signal subtracted is specified by the user. The neuropil-corrected raw fluorescence, *F*, from each neuron is used to calculate relative change in fluorescence (*F* − *F*_0_)/*F*_0 _= *ΔF*/*F*_0_, where baseline fluorescence *F*_0_ is computed from a sliding window of previous time points as described in the Materials and Methods. The baseline-corrected fluorescence traces (*ΔF*/*F*_0_) of several select neurons are then displayed in the GUI (Fig. 1).

The baseline-corrected fluorescence of each neuron serves as a proxy of its activity and is then used in further downstream analysis that elucidates the functional properties of the neuronal population. As a first demonstration of this capability, NeuroART employs a pairwise correlation-based analysis to construct functional networks and rank cells according to highest degree, explained in detail in the Materials and Methods, 2P image analysis. The downstream analysis within the real-time loop runs rapidly enough to be computed at the GUI update rate in real time on a laptop [Intel(R) Core i7-8565U CPU @ 2 GHz, 12 GB RAM, Intel UHD 620 Graphics Card, 64 bit Windows 10]. The speed of the real-time loop is limited most heavily by how many neurons are being analyzed, while a secondary rate limiter is how often the user chooses to update the GUI indicator panels (Fig. 2, middle inset). In our evaluation tests, the real-time loop processed ∼30 frames per second for 150 neurons when the GUI is set to be updated twice per second.

### Customizable downstream analysis for informed optogenetic stimulation

NeuroART implements downstream analysis as a flexible step that can integrate information from any or all prior time points, not just the current frame, mostly through analysis of *ΔF*/*F*_0_ traces that can go back arbitrary time intervals (Fig. 2, right inset). Real-time analysis of *ΔF*/*F*_0_ traces allows the experimenter to identify neuronal groups of interest: the most active or inactive neurons, the most correlated neurons, or, if used in conjunction with sensory input data and models of sensory perception, the neurons most important for sensory perception. Optogenetics have emerged as a promising tool to directly activate or inactivate individual neurons or groups of neurons and then observe how response dynamics or behavior is altered (Rickgauer et al., 2014; Grosenick et al., 2015; Gill et al., 2020). With the real-time traces obtained by NeuroART, one could achieve informed optogenetic stimulation to target neurons that fit certain desired characteristics as quantified during the experiment, enabling model-guided experiments.

While processing the *ΔF*/*F*_0_ traces and correlations in real time, some of the downstream analysis (e.g., tuning analysis, synchrony analysis) is more time intensive and thus cannot be updated as often. However, many of these downstream analyses yield quantitative descriptions of neurons that change minimally from frame to frame. Therefore, we are not limited to only including analysis that is efficient enough to be performed in every single frame but additionally to periodically conduct more intensive postprocessing analysis on the full batch of images that have been preprocessed. The time interval of this periodic analysis and the amount of information from prior time points used for it depends on the type of analysis chosen by the experimenter. For example, pairwise correlations can be computed using fewer prior time points compared with receptive field calculations which require data from multiple trials of several unique stimulus presentations as discussed in detail later.

### Integrated downstream analysis of functional connectivity and synchrony of neurons

**Figure 5.**
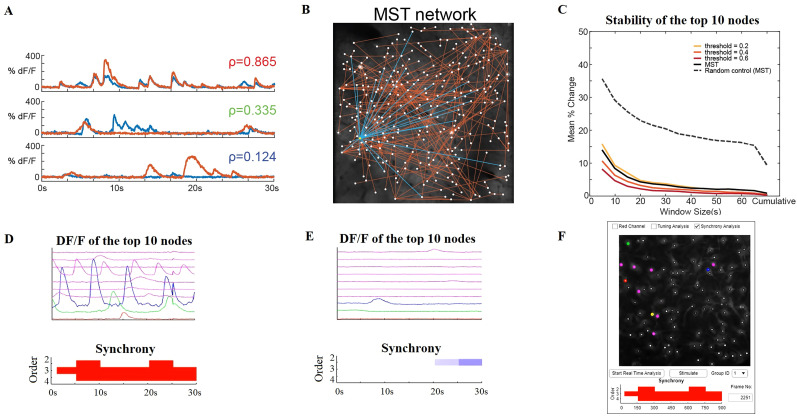
Multi-timescale downstream analysis of correlations and synchrony. ***A***, Calcium traces from three example neuronal pairs with high, moderate, and low pairwise correlations (top, middle, and bottom plots, respectively). ***B***, The functional network constructed from the minimum spanning tree (MST) technique shown in the imaging FOV. The functional connections to the highest degree neuron are highlighted in blue. The functional network is updated every second within the real-time analysis workflow. ***C***, Stability of cell ranking within the functional network depending on the size of the analysis window. The percentage change between two adjacent frames (averaged over all the frames) of the top 10 ranks is shown for each functional network type. Random control was computed on 10 surrogate datasets of random signals. ***D***, Dynamic analysis of higher-order coordination (synchrony) of neuronal spiking activity using NeuroART (for details, see Mukherjee and Babadi, 2021). Example calcium traces that demonstrate significant second-, third-, and fourth-order synchrony (only the calcium traces of the top 10 ranked cells are shown here). Figures are updated every 30 s, and a significant presence of synchrony is indicated by red color. ***E***, Example calcium traces that demonstrate significant suppression of second-order synchrony (only the calcium traces of the top 10 ranked cells are shown here). Figures are updated every 30 s, and a significant level of suppression of synchrony is indicated by blue color. In this example, there are only a few active cells, and therefore there is no coordinated neural spiking activity. ***F***, The NeuroART GUI readout for synchrony analysis. This output is displayed below the image of the augmented FOV and the user can select between different types of downstream analyses which include synchrony analysis. The top 10 ranked neurons are highlighted in the augmented FOV image where red, green, and blue represent the first, second, and third ranked neurons.

Functional connectivity of the neurons is assessed by pairwise correlations based on the covariance of the *ΔF*/*F*_0_ traces (Fig. 5*A*). NeuroART provides two methods to prune the correlation matrix into a functional network: (1) we impose a specific topology in the network or (2) we prune graph edges based on a threshold value of the correlation. In the first approach, we utilize an MST algorithm to prune the matrix down to the fewest and strongest functional connections in a manner that retains all neurons within a single network (Bowen et al., 2024; Fig. 5*B*). This allows rapid, unbiased assessment of a functional network without having to define numeric thresholds or omit neurons. In our implementation of the MST algorithm, we invert the pairwise correlation magnitudes, so that the MST retains links between the most correlated neurons (i.e., highest correlation values). In the second approach, we threshold the correlation network by omitting correlations below a value determined by the user. In the resulting networks from both methods, the degree is calculated for each neuron which indicates its overall connectivity to the rest of the network. Therefore, we can rapidly rank the population correlation of neurons in an FOV by sorting them by network degree. Using this ranking, we can target and stimulate cells and observe the effect as a function of their correlation and to the rest of the network.

NeuroART facilitates multi-timescale downstream analyses during experiments. To demonstrate this capability, we have implemented synchrony analysis of neuronal spiking activity, measuring higher-order correlations among neurons based on the framework proposed by Mukherjee and Babadi (2021). A fast online deconvolution framework (Friedrich et al., 2017) is integrated to the synchrony analysis pipeline to enable real-time synchrony analysis based on calcium activity traces. Synchrony analysis involves an adaptive greedy filtering algorithm based on a discretized Markov point process model of ensemble spiking to identify higher-order correlations in spiking activity. Due to computational constraints, we consider only up to fourth-order neuronal synchrony. During the real-time analysis workflow, the functional correlation network gets updated every second while the synchrony of spiking activity is assessed every 30 s. Synchrony is indicated in a bar graph that is distinct between periods of high synchrony (Fig. 5*D*) and periods of low synchrony (Fig. 5*E*) and is suitable for display directly in the GUI (Fig. 5*F*).

### Adjustable analysis time window

NeuroART includes the option to change the timescale of downstream analyses on the fly. By default, the real-time analysis app uses a cumulative window to analyze all acquired images for downstream functional analysis. However, the neuronal network itself may evolve during long experiments. Thus, NeuroART includes an option to pick a custom sliding window size (Fig. 1*A*, bottom left) such that real-time downstream analysis only includes a user-defined prior time window. A smaller window size will allow observation of rapid changes in correlations which might better suit an optogenetic experiment where characterization of short-term changes following a manipulation is most informative. The tradeoff is that shorter window sizes provide a less stable estimation of the state of the neuronal network. This custom sliding analysis window can be adjusted on the fly during an experiment. As one assessment of how the robustness of our analysis depends on window size, we quantified how cell rankings change with analysis window size in both MST networks and thresholded correlation networks, focusing on the top ranked neurons since those would likely be perturbation targets (Fig. 5*C*). While cumulative analysis of all available images provides the most stable correlations, network rankings remain stable down to window sizes of 30 s (Fig. 5*C*). Both MST networks and thresholded networks performed similarly with regard to the stability of cell ranking. However, thresholded networks can be very sensitive to the threshold value chosen. As a result, using the MST method is more consistent from experiment to experiment, while thresholded networks allow a wider range of choices for the sparsity of the resulting functional network.

Thresholded networks with certain threshold values may demonstrate more stability in some cases but it depends on the underlying correlations within the network. The MST method is more generalized, and it is not required to change parameters from experiment to experiment. Furthermore, thresholding correlations will ignore any weak correlations completely and omit neurons, whereas MST benefits by including every neuron with at least one connection, even if it has a weak correlation.

### Live integration of sensory metadata: auditory cortex tuning example

The ongoing development of NeuroART will involve implementing optional modules for specific use cases. As proof of concept, we have implemented a module that computes the frequency tuning of each neuron within an FOV of the auditory cortex in real time during an experiment. The typical auditory neuroscience experiment involves presenting sounds that span a range of frequencies within the animal subject's hearing range. Sounds are presented repeatedly over many individual trials (Fig. 4*A*). Depending on how neurons respond to presentations of different sound frequencies, we can assess the “tuning” of each neuron (i.e., which sound frequency is most likely to drive activity) by constructing a receptive field which averages the responses over all trials (Fig. 4*B*). The tuning module in NeuroART is optional and can be turned on and off during the experiment. The module computes the tuning of each neuron as described previously (Bowen et al., 2020) and uses information from all available trials to provide the best current assessment of tuning properties to the experimenter. The tuning module periodically updates the augmented FOV with the color-coded best frequency of each neuron (Fig. 4*C*). As a result, the experimenter can continue to add sound presentation trials and visually inspect whether the measured tuning properties are robust. Furthermore, this feature enables auditory neuroscientists to get a robust quantification of what part of the auditory cortex and tonotopic gradient they are recording from during image acquisition.

### Real-time 2P holographic optogenetic photostimulation: in vitro demonstration

NeuroART allows for tracking the activity of neurons of interest in real time. During imaging, five neurons are emphasized automatically, with *ΔF*/*F*_0_ traces displayed and updated in real time (Fig. 1*A*, middle top). As described in the Materials and Methods: Functional networks and neuronal ranking, these are the neurons that are most correlated to the activity of the rest of the population as measured by degree in the functional correlation network. The user interface provides an option to track the activity of these most correlated neurons or manually selected neurons in the FOV (Fig. 1*A*, middle bottom). While tracking the activity of the neurons of interest, the NeuroART software enables 2P holographic optogenetic photostimulation of the identified neurons for photostimulation. The coordinates in the imaging FOV are mapped to the coordinates in the SLM based on a calibration process conducted prior to the live imaging session.

To demonstrate the real-time 2P photostimulation capability (Movie 1), calcium activity (inferred through *ΔF*/*F*_0_ traces) of primary rat embryonic hippocampal neuronal cells was analyzed in real time using NeuroART (see Materials and Methods for details). These cells were transduced using the bicistronic lentiviral vector, pLV[Exp]-Bsd-SYN1-jGCaMP8s-P2A-ChrimsonR-ST, that provides robust coexpression of the calcium indicator (jGCaMP8s) and the opsin (stChrimsonR) used for holographic optogenetic stimulation (LaFosse et al., 2023). Imaging and photostimulation were performed using a custom-built dual beam path microscope (Fig. 3).

**Movie 1.** Demonstration of real-time holographic optogenetic stimulation of neuronal cells. A movie that was captured during real-time holographic optogenetic stimulation of neuronal cells. This experiment was conducted using our custom-built dual beam path microscope and the ScanImage software for image acquisition. [View online]

**Figure 6.**
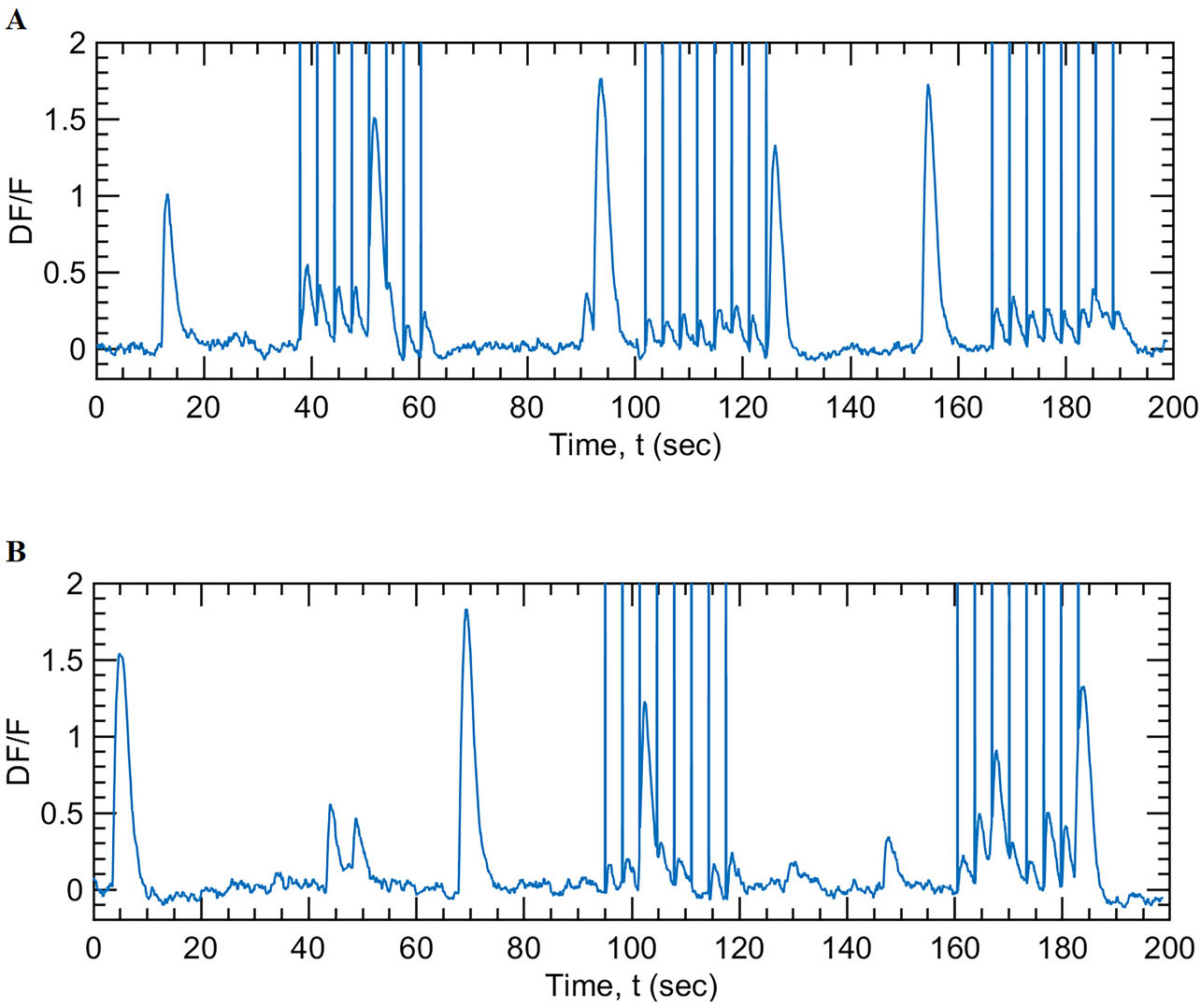
Real-time holographic optogenetic stimulation of neuronal cells. The figures illustrate the response of a single neuron during a real-time holographic optogenetic stimulation experiment conducted using a cell culture of primary rat embryonic hippocampal neurons. Optogenetic stimulation (using the photostimulation laser, 1,064 nm, 80 MHz, 160 fs, 370 mW at the sample) was provided for a duration of 30 ms, every 3 s. The vertical lines were drawn to indicate photostimulation events. ***A***, Holographic optogenetic stimulation using optical vortex-shaped beamlets of light. The three trials shown here correspond to photostimulation using optical vortices of three different sizes (vortex mode, *L* = 5, 4, and 6 respectively). ***B***, Holographic optogenetic stimulation of the same neuron using disk-shaped beamlets of light. The two trials shown here correspond to photostimulation durations of 50 and 30 ms, respectively, delivered every 3 s during photostimulation. NeuroART software facilitates adjusting these parameters (size of the beamlets, photostimulation duration, and the photostimulation frequency) in real time during an experiment.

Real-time analysis of the functional correlation network determines the neurons that are most correlated to the activity of the rest of the population. These neurons were identified as potential targets for 2P holographic optogenetic photostimulation. A separate photostimulation module is available within NeuroART to read the coordinates of the identified neurons, to calculate, and to transfer phase masks to the SLM for 2P holographic optogenetic photostimulation (see Materials and Methods for details). These projected phase masks generate holographic intensity patterns on the imaging plane of the neuronal cells that lead to spiking events (action potentials) in the targeted neurons. Subsequently, the resulting network dynamics of this perturbation can be observed in real time through NeuroART, enabling closed-loop 2P imaging and informed optogenetic photostimulation experiments. Figure 6 demonstrates how the activity of a neuronal cell (indicated by the corresponding *ΔF*/*F*_0_ traces) was modulated by NeuroART through holographic optogenetic stimulation.

The criteria used for photostimulation can be modified based on the experimental needs (e.g., if a preidentified group of cells becomes active, then automatically calling the callback function of the stimulation button in NeuroART).

## Discussion

In summary, we have developed NeuroART, software that performs real-time neuronal activity analysis during an experiment in a microscope platform-independent manner allowing for closed-loop experimental designs. NeuroART facilitates live assessment of experimental quality and integrates information from longer timescales as part of the suite of real-time downstream analyses. NeuroART demonstrates the integration of analysis tools from multiple labs into such real-time analysis. Analysis of a range of complexities are integrated in a single workflow utilizing multiple timescales, from 30 Hz measurements of calcium traces to 2 Hz figure updates, 1 Hz correlation measurements, and 0.03 Hz synchrony calculations. This enables identification of functional properties, such as the most correlated or synchronous neurons, and performing informed holographic optogenetic stimulation. The software is also well suited for intuitive on-line optimization of experimental parameters.

We have also demonstrated real-time holographic optogenetic stimulation capabilities of NeuroART through in vitro experiments involving primary rat embryonic hippocampal neuronal cells. NeuroART provides the user interface for simultaneous calcium image analysis, photostimulation, and observation of the resulting network dynamics in real time; enabling experimenters to perform closed-loop neuronal stimulation experiments.

Furthermore, as chronic imaging of awake animals has become more common, behavioral training paradigms have become more abundant. Real-time readout of neuronal activity in conjunction with behavioral variables (response latencies, hit vs miss trials) could allow a researcher to adapt their experimental conditions on the fly, based on the neuronal population activity, or ensure that there is high-quality neuronal readout to pair with behavioral readout. For example, behavioral imaging sessions typically start with a “passive” block to get a baseline reading of neuronal activity prior to starting the “active” block where behavioral tasks are performed. Real-time analysis during a passive block could streamline this process and ensure that the imaging FOV will produce long-term consistent and high-quality neuronal population recordings prior to starting the full behavioral paradigm experimental block. In our first demonstration of this approach, we identify neurons during a passive block and subsequently infer tonotopy for each of the identified neurons.

This integration of sensory stimulation information, e.g., to quantify tuning preference of neurons, is one of the unique capabilities of NeuroART. When compared with other recently developed software (Giovannucci et al., 2017; Mitani and Komiyama, 2018; Zhang et al., 2018; Giovannucci et al., 2019; Sheng et al., 2022) in this growing area, as reported in Table 2, NeuroART also enables real-time inclusion of functional network analysis, correlation analysis, and real-time holographic optogenetic stimulation driven by knowledge gained from real-time analysis. With these unique features and its demonstrated ability to work with several widely used microscope platforms, NeuroART is poised to enable novel closed-loop model-guided experiments.

**Table 2.** Comparison of NeuroART to other available software tools for real-time analysis of neuronal activity: CaImAn Online (Giovannucci et al., 2019), OnACID (Giovannucci et al., 2017), Vidrio ScanImage 2021, ClosedLoop (Zhang et al., 2018), Mitani et al. (Mitani and Komiyama, 2018), and ORCA (Sheng et al., 2022)

	NeuroART	CaImAn ONLINE (2019)	OnACID (2017)	ScanImage (2021)	ClosedLoop (2018)	Mitani et al. (2018)	ORCA (2022)
Motion correction	✓	✓	✓	✓	✓	✓	✓
Automated cell identification	✓	✓	✓	X	✓	X	✓
Fluorescence extraction	✓	✓	✓	✓	✓	✓	✓
*ΔF*/*F*_0_ calculation	✓	✓	✓	X	✓	✓	✓
Functional network analysis	✓	X	X	X	X	X	X
Multi-timescale analysis (e.g., correlations, synchrony)	✓	X	X	X	X	X	X
Identification of stimulation targets	✓	✓	X	X	✓	X	✓
Real-time holographic optogenetic photostimulation	✓	X	X	X	X	X	X
Integration of sensory information	✓	X	X	X	X	X	✓
Works on multiple imaging and operating systems (OS)	✓	✓	X	✓	X	X	✓

## Synthesis

Reviewing Editor: Arvind Kumar, KTH Royal Institute of Technology Decisions are customarily a result of the Reviewing Editor and the peer reviewers coming together and discussing their recommendations until a consensus is reached. When revisions are invited, a fact-based synthesis statement explaining their decision and outlining what is needed to prepare a revision will be listed below. The following reviewer(s) agreed to reveal their identity: Riichiro Hira, Benjamin Scholl.

Synthesis

The two reviewers have agreed that NeuroART can be a useful software for real-time analysis and holographic simulation experiments. The software can be particularly valuable in closed-loop experiments. The reviewers have raised several questions that need to be addressed before we can make a final decision. One of the reviewers tried to install and work with the toolbox in her lab. But she/he had a hard time getting the software to work. This is of course not very encouraging. Without assessing the software during live acquisitions, it is impossible to review this work.

We recommend that the authors make the software easy to install and work with. In addition, it would help if authors could find some labs to be beta-testers. Showing their system being used by another lab and conducting experiments (even just examples) would be immensely valuable and would help work out all the kinks in the software.

Detailed comments from the reviewers are appended below.

Reviewer#1

This article presents NeuroART, software that allows real-time analysis of neural activity and holographic photostimulation of neurons. NeuroART can perform downstream analysis of functional connectivity, correlation, and synchrony with various parameters adjustable on the fly. NeuroART allows users to identify Regions of Interest by either manually clicking, loading previously identified cell coordinates, or automatically via CaImAn. NeuroART is also equipped with an optional module that analyzes the frequency tuning of each neuron in the auditory cortex and further development of live integration of sensory metadata is in progress. In addition, NeuroART allows holographic optogenetic photostimulation of identified neurons by automatically mapping the coordinates of imaging FOV to that of SLM, enabling closed-loop stimulation experiments. The wide variety of real-time analysis, integration of sensory stimulation information, and informed holographic optogenetic stimulation as well as its compatibility with several widely used imaging systems distinguishes NeuroART from other recently developed software.

Due to the importance of the closed-loop experiments using two-photon microscopy in this field, I recommend acceptance of this manuscript for publication in eNeuro after appropriate revision. As pointed out below, the authors need to somewhat refrain from asserting the usefulness of NeuroART's especially on the correlation analysis and on the integration of SLM. Table 1 shows a comparison with previous studies, and many of the advantages claimed by the authors are trivial compared to the realization of a real-time system itself. The correlation analysis may not be reported with other tools, but such analysis can easily be added, and there is no element of NeuroART being a system specifically capable of doing so. Also, the SLM integration is described as real-time, but there is no demonstration to say it is done at real-time.

1. For NeuroART to integrate imaging software such as Thorlabs, ScanImage, or Praire view, is it necessary to modify the scripts (global variables, etc.) on the part of the imaging software? If so, please provide specific examples of how they were modified. Or, does the NeuroART read the data written to the SSD each time? If so, please indicate the read/write speed of the SSD. As for software from Leica, Zeiss, Nikon, Olympus, etc., I believe they store their images in proprietary formats, making it difficult to read the data from Matlab. If so, an explanation should be added that it is difficult to use with these common two-photon microscopes. Alternatively, it would be good to mention whether real-time analysis is possible in such cases, for example, by branching the PMT signal or by transferring the image displayed on the monitor to the other computer.

2. Line 242-: When can the claim be made that 2P holographic optogenetic photostimulation is real-time? In the authors' strategy, target cells can be identified during imaging. For that set of target cells, the GS algorithm could probably successfully generate SLM patterns. The authors have demonstrated that the SLM patterns can be used to stimulate the target cells. However, I think we say that this process is real-time only if this stimulation can be triggered by some event. It could be, for example, the moment a particular group of cells is activated or the moment the animal makes a decision. If this is a possible setup, please provide specific examples. Otherwise, the claim that real-time holographic stimulation is possible lacks validity. Simply having a module that can do CGH is not real-time.

3. Lin 318-: The hologram generation is done by the GS algorithm, but the time taken to derive the SLM pattern by the GS algorithm should be specified. It should also be clearly stated whether the pattern optimization requires feedback on the acquired images. If it is required, then on-the-fly as well as real-time stimulation is not possible, since multiple trials of non-optimized stimulation are required.

4. Line 326: Regarding the sequential mode in which SLM patterns are prepared for pre-designated target cells, is it possible to respond in real-time to the inevitable parallel shift of the cell position in the XY or Z direction within the field of view when awake animals are the target? If not, will this need to be resolved before moving from in vitro demonstrations to in vivo experiments? Especially if you want to claim that you can stimulate in real time.

5. Line 312: Please add the repetition rate of the laser (MHz) and the required laser power (mW/cell) to stimulate.

6. Throughout the experiment, images were registered using discrete Fourier transform registration in MATLAB. While it may be applicable for small fields of view, in the case of wide-field imaging, it is more appropriate to use piecewise-rigid (pw-rigid) registration, which is also done in NoRMCorre in CAIMAN. Is it possible to use this method of registration, and if so, how would it affect the real-time loop benchmark on NeuroART?

7. Line: 222: "However, thresholded networks can be very sensitive to the threshold value chosen." However, fig3 C does not show a large variance in stability with respect to the threshold value. Rather, a threshold above 0.4 demonstrates more stability over the MST method.

8. In Figure 2, the box that says "Update correlation networks and cell degree ranking" should be placed outside the black box indicating a loop at 30Hz for 100 neurons, since correlation measurements occur only once every second, according to line 415.

Reviewer #2

This manuscript describes NeuroART, a software tool that integrates real-time analysis of neuronal activity with optogenetics for targeted brain stimulation. The authors suggest that NeuroART can provide live readouts of neuronal activity and analyze several functional propoerties of neural activity (e.g. correlations). It reportedly operates independently of existing microscope software, supporting various microscope platforms and facilitating collaboration among research groups. The software is designed to control a Spatial Light Modulator to execute holographic photostimulation, enabling closed-loop experiments.

We have significantly struggled with the software package. After we received it, it was relatively easy to install, although none of our imaging machines contained a sufficient graphics card for GPU-computing. We were able to follow the instructions and code to initialize an experiment, but were not able to get the "Live" function working. We were unsuccessful on all of our microscope systems. We really do not know if this is a bug in the code or a user error, but after attempting to get the software working for more than 1 day in the lab, we have given up. Without assessing the software during live acquisitions, it is impossible to review this work.
